# Impact of Solar Radiation on Gene Expression in Bacteria

**DOI:** 10.3390/proteomes1020070

**Published:** 2013-07-16

**Authors:** Sabine Matallana-Surget, Ruddy Wattiez

**Affiliations:** 1 UPMC Univ Paris 06, UMR7621, Laboratoire d’Océanographie Microbienne, Observatoire Océanologique, Banyuls/mer F-66650, France; 2 CNRS, UMR7621, Laboratoire d’Océanographie Microbienne, Observatoire Océanologique, Banyuls/mer F-66650, France; 3 Department of Proteomics and Microbiology, Research Institute for Biosciences, Interdisciplinary Mass Spectrometry Center (CISMa), University of Mons, Mons B-7000, Belgium; E-Mail: ruddy.wattiez@umons.ac.be

**Keywords:** transcriptomic, proteomic, gene regulation, radiation, bacteria

## Abstract

Microorganisms often regulate their gene expression at the level of transcription and/or translation in response to solar radiation. In this review, we present the use of both transcriptomics and proteomics to advance knowledge in the field of bacterial response to damaging radiation. Those studies pertain to diverse application areas such as fundamental microbiology, water treatment, microbial ecology and astrobiology. Even though it has been demonstrated that mRNA abundance is not always consistent with the protein regulation, we present here an exhaustive review on how bacteria regulate their gene expression at both transcription and translation levels to enable biomarkers identification and comparison of gene regulation from one bacterial species to another.

## 1. Introduction

Bacteria present a wide diversity of tolerances to damaging radiation and are the simplest model organisms for studying their response and strategies of defense in terms of gene regulation. There is a broad range of application areas justifying the study of bacterial response to radiation ranging from fundamental microbiology, microbial ecology to better predict the impact of future climate change with the ozone hole, UV-decontamination for water treatment, astrobiology for future manned missions in space and industrial applications (such as cosmetics). Sunlight produces a broad spectrum of radiation from which a part reaching the Earth’s surface is composed of the UV radiation (UVB, UVA), visible light and infrared, while space radiation is different from the kinds of radiation we experience on Earth, including the ionizing radiations such as gamma rays. Exposure of microorganisms to solar radiation leads to direct and indirect damage to the cell. 

Nucleic acid is a key component of radiation-induced cellular damage. The UVB fraction induces dimerization of pyrimidine bases, leading to the formation of two major photoproducts, cyclobutane pyrimidine dimers (CPDs) and pyrimidine (6-4) pyrimidone photoproducts (6-4 PPs) [1,2]. A total of 12 photoproducts can be induced but they are not all produced with the same frequency and mainly differ according to the GC content of the prokaryotic genomes [3]. Ionizing radiation leads to severe DNA/RNA damage such as double/single strand breaks, base modifications [2,4,5]. UVA and visible light can lead to detrimental oxidative stress by generating reactive oxygen species (ROS) that interact with DNA, proteins and lipids, or might have a beneficial action by activating a specific light-dependent repair enzyme (photolyase) involved in a mechanism called photoreactivation [1,2]. The resulting DNA lesions generated by oxidative stress include base and sugar lesions, strand breaks, DNA-protein cross-links and base-free sites [6,7,8,9]. 

**Figure 1:**
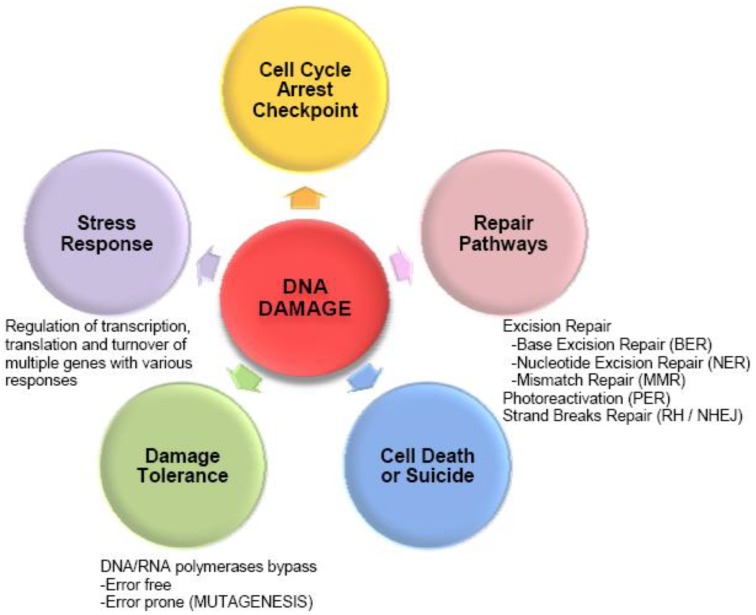
Biological responses to DNA damage.

The consequences of DNA lesions are either an inhibition of the progression of the polymerase during DNA replication and transcription or a lesion bypass with misincorporation that could eventually lead to mutations [1,2] (Figure 1). Similarly, strand breaks or oxidative damage to protein-coding RNAs or non-coding RNAs might cause errors in protein synthesis or disregulation of gene expression [4]. To protect from cell impairment, microorganisms have a wide variety of strategies to reverse, excise, or tolerate DNA damage (for a review see [1,2]) (Figure 1). A general response to DNA damage is a delay or arrest of the cell cycle providing more time for DNA repair. Checkpoints were actually first described in bacteria, although the same terminology was not used when they were (see review [10]) (Figure 1). The suicide response predicts that rapidly growing and respiring cells will suffer growth arrest when subjected to relatively mild stresses, but that their metabolism will continue. A burst of free radical production results from this uncoupling of growth from metabolism and it is this free radical burst that is lethal to the cells, rather than the stress per se [11] (Figure 1). The net biological effect of damaging radiation depends upon the balance between the rate of radiation-induced damage and both the efficiency of how the cell protects itself against damage accumulation as well as the rate at which that damage is repaired. However some damage are not repaired in the cell such as oxidative proteins and lipids damage and the level of accumulation of protein damage in the cell plays a pivotal role in bacterial radioresistance [12,13] and until now, have been much less studied than DNA damage. 

Transcriptomic and proteomic approaches are well adapted for studying the overall gene regulation of bacteria exposed to a radiative stress. To date a wide range of methods exists to characterize the changes in the transcriptome and proteome of bacteria. Among the several transcriptome profiling methods, microarray and RNA-seq are the two most popular methods employed for studying gene expression and regulation of mRNA synthesis under damaging radiation and/or repair conditions. While microarray has been extensively used as transcriptomic approach, today the RNA-seq method is becoming a preferred method [14,15,16,17,18], mainly because it does not depend on the previous characterization of the reference transcriptome. In the present review focusing on the particular radiation stress, almost all the studies used the microarray method and only one recent work used the RNA-seq approach [17]. Although it is known that there is not necessarily a correlation between the microarray results and proteomics, it has been reported that mRNA expression measurements made with the RNA-seq technology better correlate with proteomic data than microarrays [18]. Protein expression mapping has an advantage over monitoring mRNA levels in that it is a direct measure of the protein product of a gene. A wide range of mass spectrometry-based approaches for quantifying proteins are available today, these can be gel-based or gel-free approaches. Two-dimensional gel electrophoresis (2D-DIGE) allows the routine separation of thousands of proteins, and the simultaneous detection and quantification of paired samples on a single gel and has significantly decreased gel-to-gel variability by using a single gel for cross sample comparison [19]. Today, stable isotope labeling is the most comprehensive gel-free approach for measuring overall protein abundance and can be performed by *in vitro* (ICAT, iTRAQ, ICPL post-digest) and *in vivo* (e.g., metabolic labeling) approaches [20,21,22,23,24]. There are some problems with using ICAT for comparative expression studies in response to radiation conditions. Several amino acids are prone to oxidation upon solar radiation and cysteine can be modified by damaging radiation to form oxidized cysteine sulfhydryl residues, preventing ICAT labeling. Gel-free studies on the impact of solar radiation will have to use another kind of targeting labeling such as the amine-reactive tagging reagents available with the iTRAQ technology or ICPL post digest, that yield the N-terminus and side chain amines of peptides from tryptic digests. A disadvantage of *in vitro* labeling such as iTRAQ or ICPL is that the stable isotope label is introduced into the sample only after several stages of sample preparation, such as cell lysis, protein extraction and proteolysis. Therefore, it is usually preferred to introduce the stable isotope label very early in the process, such as in the metabolic labeling approach. By using this method, protein can be metabolically labeled by growing cells in either 14N minimal or 15N-enriched media, as the sole nitrogen source that can be incorporated during protein synthesis [25]. This technique provides a complete proteome coverage in which all peptides are labeled. So far, metabolic labeling has been applied mostly to unicellular organisms, such as yeast [26] and bacteria [27], which can be easily grown on defined media in the laboratory. Due to the development of high sensitive mass spectrometers, it is now possible to get rid of the labeling step to perform directly label-free quantitative proteomics. To date, less quantitative proteomics studies have been reported on the impact of damaging solar radiation compare to transcriptomic approaches (Table 1). 

**Table 1:** Studies related to gene expression change following treatment to radiation in bacteria.

Microorganism	Radiation	Gene Regulation	Application areas	References
*Deinococcus gobiensis*	Gamma radiation + UVC radiation	Transcriptome	Resistance analysis	[17]
*Deinococcus radiodurans*	Ionizing radiation	Transcriptome	Resistance analysis	[28,29]
		Proteome		[30]
		Proteome	Proteogenomics/ Resistance analysis	[31]
*Nostoc commune*	UVB radiation	Proteome	Resistance analysis	[32]
*Deinococcus deserti*	UV radiation, Ionizing radiation	Proteome	Proteogenomics	[33]
*Ruegeria pomeroyi* DSS-3	Ionizing radiation	Proteome	Proteogenomics	[34]
*Shewanella oneidensis* MR-1	UVA, UVB, UVC	Transcriptome + Proteome	Proteogenomics	[35]
		Transcriptome	Sensitivity analysis	[36]
	Ionizing radiation	Transcriptome	Sensitivity analysis	[37]
*Escherichia coli*	UVA	Transcriptome	Bacterial Disinfection	[38]
*Bacillus pumilus*	“UV-Mars” “UV-Space”	Proteome	Astrobiology	[39]
*Rhodospirillum rubrum*	Ionizing radiation	Transcriptome/ Proteome	Astrobiology	[40]
*Prochlorococcus* *marinus* MED4	High light intensity	Transcriptome	Microbial ecology	[41]
		Proteome		[42]
*Synechocystis* sp. strain PCC 6803	High light intensity + UVB	Transcriptome	Microbial ecology	[43,44]
*Photobacterium angustum* S14	UVB	Proteome	Microbial ecology	[45]
*Sphingopyxis* *alaskensis* RB2256	UVB, UVA, Visible light	Proteome	Microbial ecology	[46]

In this review, we present the main results of transcriptomic and proteomic, analyzing the modification of gene expression in bacteria exposed to different radiation sources, applied to multiple applications areas. We also present the studies focusing on post-translational studies and especially protein damage such as protein carbonylation, that influence protein turnover and thus protein abundance, a key stone when performing quantitative proteomics following radiative stress.

## 2. Understanding the Extreme Radiation-Resistance of Key Bacterial Model

One goal of photobiology is to understand and explain why cells are very resistant to damaging radiation. One bacterium best known for its extreme resistance to the lethal effects of ionizing radiation is the aerobic gram-positive *Deinococcus radiodurans*. Two studies focused on the transcriptome of *D. radiodurans*, using different physiological growth states (exponential *vs.* stationary phase) and different doses of γ radiation (3 kGy *vs.* 15 kGy) [28,29] (Table 1). While the first study performed by Liu and co-workers in 2003 [28] reported that 832 genes were induced during a 24-h recovery period in *D. radiodurans* cultures exposed to 15 kGy γ-radiation, this high number was significantly decreased in the study of Tanaka and colleagues [29] with only 72 genes over expressed within the first hour after exposure to a 3 kGy γ-radiation. This difference is mainly due to the different experimental designs of both studies as well as the different level of stringency used for data analysis with a threshold of either twofold [28] or threefold [29] for validating expression changes. The function of most regulated genes under ionizing radiation was found to be either similar to other genes commonly identified in other bacteria exposed to damaging radiation or functionally uncharacterized [28,29]. Similarly, a recent work on another radioresistant bacterium *Deinococcus gobiensis* using high resolution RNA-Seq technology to analyze the UVC-induced transcriptome showed an important fraction of unknown genes function and pathways [17] (Table 1). As it was found in the study of *D. radiodurans*, genes involved in the photoreactivation (*phrB*) and recombination repair (*recB*) were found to be induced immediately after UV exposure and thus remain a key player in its resistance phenotype [17]. A quantitative proteomic approach with 2D gels was used to compare the proteomes of irradiated and unirradiated *D. radiodurans* bacteria to identify the mechanisms of its extreme radioresistance and DNA repair [30] (Table 1). Only 26 protein spots showed significant changes between both conditions of irradiation and 21 proteins were correctly identified by mass spectrometry. Most of these proteins had cellular functions such as (i) translation; (ii) transcription; (iii) signal transduction; (iv) post-translational modification, protein turnover, chaperones; (v) carbohydrate transport and metabolism; (vi) energy production and conversion; (vii) nucleotide transport and metabolism; (viii) inorganic ion transport and metabolism, but none of them was known to be relevant to radioresistance, except for the single-stranded DNA-binding protein (SSB) and PprA proteins, that was previously found to be critical for the radiation resistance of *D. radiodurans* as an enhancer of DNA ligation [47]. In contrast to the previous transcriptomic approaches [28,29], the RecA protein was not identified [30]. Indeed, it is important to point out that the proteomics study using 2D gels only characterized a very few number of proteins, that do not allow us to perform a substantial comparison between the transcriptomic and proteomic studies. Thus, proteomics did not enable to clearly understand the extraordinary resistance of *D. radiodurans* towards damaging radiation [30]. However, the study of Tanaka and co-workers [29] performing several mutants (*ddrA*, *ddrB*, *ddrC*, *ddrD*, and *pprA*) of *D. radiodurans*, demonstrated that part of the explanation for its radioresistance was also found among proteins of unknown function and not only linked to the efficient DNA repair pathway involving the recA enzyme. These unknown proteins appeared to mediate efficient RecA-independent processes connected to IR resistance [29]. 

The proteome of the cyanobacterium *Nostoc commune*, a dessication-tolerant terrestrial cyanobacterium was analyzed during continuous growing culture under UVB radiation using 2D gels. 493 protein spots showed significant changes (at least by a factor three compare to their corresponding unirradiated growing culture), rendering the UVB stimulon of *Nostoc commune* the most complex one described to date [32] composed of two types of response: an early shock response influencing 214 proteins and a late acclimation response involving 279 proteins, with no or few overlaps between the two responses. The shock response involved many membrane proteins, whereas the acclimation response mainly affected cytosolic proteins. UV irradiation induced superoxide dismutase and the water stress protein in the extracellular fraction. A total of 27 UVB-induced proteins were partially sequenced by mass spectrometry. They were mainly involved in lipid and carbohydrate metabolism and in regulatory pathways. As much as 50% of the sequenced proteins of the UVB acclimation response remained uncharacterized, with no known function. This study supports the idea that short-term stress and acclimation responses to damaging radiation are two completely different and remarkably complex strategies [32].

Today, proteomic approaches can be also used to verify coding regions of a genomic sequence, and are thus developed as an aid for the genome annotation, especially for bacteria that provide a special feature of interest such as radioresistance. This emerging field, called proteogenomics [48], was used to re-analyze the genomes and decipher the proteomes of the following bacteria: *Deinococcus radiodurans* [31], *Deinococcus deserti* [33], *Ruegeria pomeroyi* DSS-3 [34], *Shewanella oneidensis* [35] (Table 1).

## 3. Sensitive *vs.* Resistant Bacteria

While *Deinococcus radiodurans* can survive levels of ionizing radiation (10 kGy) that induce a hundreds of DNA double strand breaks per genome, *Shewanella oneidensis* is killed by levels of ionizing radiation (0.07 kGy) that result in less than 1 double strand break per genome [49]. The comparison of the IR-responses of sensitive *vs.* resistant bacteria should enable to identify the factors peculiar to radiation-sensitive and radiation resistant phenotypes. In this purpose, the response of the radiation sensitive bacterium *S. oneidensis* is presented in this section as well as its comparison with the resistant bacterium *D. radiodurans*.

Two transcriptomic studies have been performed on *S. oneidensis* to elucidate its great sensitivity to different kind of radiations [36,37] (Table 1). This sensitivity could not be explained by its genome, which is very similar to that of *E. coli*, with the major DNA damage repair/tolerance systems, including SOS response, recombination repair, mutagenic repair, nucleotide excision repair, mismatch repair and a DNA photolyase [50]. Qiu and colleagues [36] first investigated and compared the transcriptomic responses to UVC, UVB and UVA. The authors found that *S. oneidensis* MR-1 expressed twice as many genes after exposure to UVA light than after UVC treatment. This result points to the UVA light-induced stress response being very complex and that different wavelengths interfere with different molecular targets [13]. Although the SOS response was observed with all three treatments, the induction was more robust in response to short wavelengths. Similarly, more prophage-related genes were induced by UVC and to a lesser extent UVB. The synthesis of antioxidant enzymes and iron sequestering proteins were increased in response to UVA, enabling the cell to scavenge reactive oxygen species, such as ferritin like protein Dps, TonB dependent receptor, bacterioferritin and ferrochelatase. Indeed, the intracellular concentration of iron can have a detrimental effect in near UV radiation. Iron containing proteins may act as chromophores, becoming excited and thereby being damaged directly. Hence, the regulation of iron uptake and metabolism and iron sequestration are important protection mechanisms against UVA induced oxidative damage. The relation between transition metals and UV damage in bacteria has been addressed by Santos and co-workers [51]. Contrasting results were obtained when the same bacterial strains were exposed to natural sunlight [36]. As previously observed under artificial UVA lamps, the authors observed an induction of DNA damage-repair genes, the SOS response and detoxification strategies [36]. However, a greater number of genes involved in detoxification were induced by natural solar radiation than by UVA lamps. No prophage gene was found to be induced by natural solar radiation. This suggests that the biological effects induce by the whole spectrum of natural solar radiation are not the simple sum of UVA and UVB effects. The broader transcriptomic response to natural sunlight could be due to synergistic effects of various UV wavelengths or possible effects induced by visible light. Finally, *S. oneidensis* was exposed to ionizing radiation (IR) and its transcriptome analyzed [37]. IR is often used to produce reactive oxygen species. The genomic response of *S. oneidensis* to IR was very similar to its response to UVA and UVC radiation, with induction of genes encoding antioxidant enzymes, systems involved in DNA repair, prophage synthesis and the absence of differential expression of tricarboxylic acid cycle activity [47].

If we compare the different IR-induced responses of the very sensitive bacterium, *S. oneidensis* with those of the highly resistant *D. radiodurans* we should be able to identify the factors peculiar to radiation resistant and radiation-sensitive bacteria. A prominent feature of the transcriptome IR-response of *D. radiodurans* is the differential regulation of its TCA cycle, where the isocitrate-to-fumarate steps of the TCA cycle are repressed and the glyoxylate bypass genes strongly induced by irradiation [28]. Isocitrate lyase converts isocitrate to succinate and glyoxylate, allowing the carbon that enters the TCA cycle to bypass the formation of α-ketoglutarate and succinyl coenzyme A. It has been proposed that such regulation might strongly suppress oxidative stress in *D. radiodurans*, perhaps as a mechanism for preventing additional loss of genome integrity [28]. The response of *S. oneidensis* showed no significant change in its TCA cycle, including its glyoxylate bypass. On the other hand, unlike *D. radiodurans*, *S. oneidensis* showed early induction of catalase, maybe the result of greater oxidative stress generated in *S. oneidensis* during irradiation [28]. RecA protein which is central to genomic restoration after irradiation, was found to be substantially up-regulated subsequently to ionizing radiation as well as genes involved in DNA replication, repair, and recombination, cell wall metabolism, cellular transport, and many encoding uncharacterized proteins, demonstrating that this bacterium efficiently coordinate its recovery by a complex network, within which both DNA repair and metabolic functions play critical roles. *D. radiodurans* and *S. oneidensis* appear to use different strategies to fight against oxidative stress following IR. This comparison of the two bacteria seems to better explain the high sensitivity of *S. oneidensis* than the strong resistance of *D. radiodurans*. 

## 4. Bacterial Disinfection for Water Treatment and Astrobiology Applications

The response of enteric bacteria, particularly *E. coli*, to solar UV light has been well investigated for more than 60 years [52]. Exposure to solar radiation is a common method used for bacterial disinfection for water treatment. The growth of *E. coli* is inhibited by continuous UVA radiation with a subsequent adaptation to stress [38]. Transcriptomic approach was used to assess short-time stress and UVA light adapted growth. More genes (*i.e.*, 312) were expressed in the cells irradiated for a short time (1 h) than in UVA-adapted cells (50 h) (*i.e.*, 193) (Table 1). The number of up- and down-regulated genes was almost the same for both times of irradiation. The results indicate that UV-induced the up-regulation of the synthesis of several amino acids, such as valine, leucine, isoleucine, phenylalanine, histidine and glutamate, suggesting a possible direct inhibition of enzymes involved in the uptake or/and inhibition of N-assimilation. The findings of Berney and co-workers [38] corroborate earlier reports on the induction of the SOS response in UVA-irradiated cells [53]. The induction of genes such as those encoding recA, recN, dinD, dinl and UmuD, strongly points to DNA damage as a result of exposure to UVA light. In addition, the involvement of oxidative stress was confirmed with the induction of alkylhydroperoxidase reductase, the enzyme that converts lipid hydroperoxides to their corresponding alcohols. The RpoS gene was unexpectedly repressed in the light-adapted cells. This gene encodes a global stress regulator; several acid stress resistance genes were also significantly down-regulated. This latter repression might be the result of shutting down unneeded biosynthesis [38]. The decontamination with UV can also be used to remove terrestrial bacteria associated with spacecraft to avoid taking a risk for further bacterial contamination, in order to maintain the scientific integrity of life detection for future space missions. A quantitative proteomics study used the bacterium *Bacillus pumilus* that exhibited high resistance to decontamination techniques [39] and showed that the superoxide dismutase, outer spore coat protein A, glyceraldhyde-3-phosphate dehydrogenase, transcriptional regulator, ArbB family played a key role in conferring resistance traits to this bacterium [53] (Table 1). Another study, related to astrobiology application, focused on the response of the bacterium, *Rhodospirillum rubrum* S1H, to ionizing radiation. The European Space Agency through the MELiSSA (Micro-Ecological Life Support System Alternative) project aims at recycling the organic waste produced by the astronauts into oxygen, water and food using a loop of bacterial and higher plant bioreactors [54]. *R. rubrum* is one of the key players involved in this artificial microecosystem which supports life in space, and understanding its response to space flight is a key stone. In that purpose, *R. rubrum* S1H was exposed to space-ionizing radiation and both transcriptomic and proteomic were performed. It was demonstrated that a low dose of ionizing radiation (2 mGy) could induce a significant response at the transcriptomic level, although no change in cell viability and only a few significant differentially expressed proteins were observed [40] (Table 1). 

## 5. Response of Marine Bacteria to Solar Radiation: A Case Study in Microbial Ecology

The oceans are estimated to contain more than 10^29^ bacteria [55], where those microorganisms are fundamental components of the aquatic biogeochemical cycles. Solar ultraviolet radiation (UVR, 280–400 nm) has been shown to reach significant depths in many marine ecosystems, influencing a large part of the surface of the water column, where phytoplankton productivity takes place [56]. Marine bacteria present at the surface of oceans are exposed to the full spectrum of solar radiation. Both UVB and UVA can have important detrimental effects on bacterial activity, phytoplankton photosynthesis and photochemical transformation of dissolved organic matter. Lately, environmental changes related to the depletion of the stratospheric ozone layer [57] raise concerns about the response of aquatic microorganisms that could be significantly altered by the increasing level of damaging UVR.

Three studies focused on cyanobacteria and their adaptation to different light intensities or UVB radiation (Table 1). One used the marine cyanobacterium *Prochlorococcus marinus* MED4 that has the most compact genome of all free-living photoautotrophs (1716 protein-coding genes) and the peculiarity of being adapted to an oceanic environment with low nutrient and high light intensities [41]. A transcriptomic approach was used to explore the pattern of responses to different light qualities and intensities. The expressions of approximately 10% and 5% of all genes were significantly altered by at least 2-fold, when shifted from darkness to high white light and white light, respectively. Not surprisingly, high-light had the most dramatic effect on gene expression. The most differently expressed group of genes belonged to the high-light-inducible genes (hli). The most highly up-regulated gene (61.2-fold up-regulated upon darkness-to-high-light transition) unfortunately encoded a protein of unknown function, as did the most highly down-regulated gene (11.3-fold reduced in high light). Although seven different conditions were tested, only blue light elicited a strong response. Bacterial cryptochromes seem to be good candidates for the blue-light sensors, since the majority of known light-sensing proteins are absent from its genome [41]. A quantitative proteomic approach using iTRAQ was performed on the same strain of *P. marinus*, grown under three light intensities (low, medium and high) [42]. Approximately 11% of the proteome was identified. 15 proteins were deemed to be significantly influenced by changing light intensities, particularly the down-regulation of photosystem-related proteins, and the up-regulation of the stress related chaperones in high light compared to low light treatment. Six stress related proteins were identified: GroEL, DnaK, HtpG, ClpC, and GroES. GroEL, HtpG, and GroES were deemed to be significantly up-regulated in response to high light intensities. Chaperonin proteins prevent cell damage by preventing the accumulation of misfolded or unfolded proteins that have lost their function. Transcript levels of these proteins were up-regulated in *P. marinus* MED4 in a study using quantitative RT-PCR [58] but were not discussed in the transcriptome study of Steglich and co-workers in 2006 [41]. A transcriptomic study performed on *Synechocystis* sp. PCC6803 on the impact of high light intensity also described high transcript levels of GroEL and GroES proteins after high light exposure [43].

A transcript profiling methodology was used to elucidate the expression patterns of the cyanobacterium *Synechocystis* sp. strain PCC 6803, in order to investigate changes in gene expression induced by irradiation with UVB and high-intensity white light. Several families of transcripts were found to be altered by both high intensity white light and UVB, with a subsequent down-regulation of the genes involved in the light-harvesting system, photosynthesis, photoprotection, and the heat shock response [44]. These two profiles comparisons also corroborated the regulation of many pathways, including the synchronized induction of D1 protein recycling and a coupling between decreased phycobilisome biosynthesis and increased phycobilisome degradation. However, the gene expression profiles produced by high-intensity white light and UVB differed mostly in the regulation of several transcriptional processes, and in the regulation of the ribosomal protein transcripts, which are only repressed by UVB radiation [44]. The transcriptome regulation obtained upon intense white light irradiation in Huang and co-worker publication confirmed the profiles described earlier by Hihara *et al.* performed under similar conditions [43]. 

A recent study has highlighted the key differences in the proteome regulation of two heterotrophic marine bacteria, adapted to different oceanic environments such as *Photobacterium angustum* a copiotrophic bacterium, specialized for life in nutrient-enriched habitats of the sea, while *Sphingopyxis alaskensis*, oligotrophic bacterium is adapted to life in low-nutrient environments [45] (Table 1). Even though we would have expected to observe a greater level of resistance in oligotrophic bacteria, which are hypothetically better adapted to cope with UVB than copiotrophic prokaryotes, we observed a response that was more subtle. We demonstrated that *P. angustum* and *S. alaskensis* may have very distinct strategies for resistance to UV radiation. Although *S. alaskensis* presented a very efficient photoenzymatic repair system, no significant evidence of overexpression of proteins directly involved in DNA repair (*i.e.*, photolyase) was observed in the proteomics approach. In contrast, the RecA protein was found to play a key role in the UVB resistance of *P. angustum* and characterized as one of the UVB biomarkers. Both bacteria showed an active response regarding oxidative stress defense, and several key proteins, including thioredoxin/glutaredoxin, glyoxalase, glutathione, the antioxidant Ahpc, and several chaperonin proteins (DnaK, GroES/EL, and the trigger factor) were up-regulated. When *S. alaskensis* was treated with UVB, iron homeostasis appeared to play an important role in its survival [46], similarly as it was observed in the sensitive bacterium *Shewanella oneidensis* [36]. A significant qualitative difference in the regulation of proteins abundance in *S. alaskensis* was observed between a short and long time of irradiation to artificial sunlight (UVB, UVA, Visible light) as it was already described in *Escherichia coli* and *Nostoc commune* [32,38]. However, extending the duration of irradiation exposure of stationary-phase cultures did not increase the total number of quantitative changes [46]. Visible light and UVA radiation had a major impact on the expressed proteome of *S. alaskensis* without greatly affecting viability and protein synthesis, underscoring the importance of the molecular responses for maintaining growth and survival. *S. alaskensis* may have selected a strategy of protection as opposed to the efficient strategies for DNA damage removal and replacement of cellular components selected in *P. angustum* [45,46]. 

## 6. UV-Induced Protein Damage

It is noteworthy that the abundance of proteins can change not only as a result of gene expression, but also by increasing/decreasing protein stability and turnover, that can be in turn modulated by the level of proteins lesions. Proteins are important targets of damaging radiation and it seems that the ability to protect proteins against oxidation distinguishes radiation resistant bacterial species from radiation sensitive ones. Solar radiation can generate a wide range of protein damage due to oxidative stress, such as amino acid modifications, carbonyl group formation, fragmentation, formation of protein-protein cross-links, and formation of S–S bridges. A recent review presented modifications induced by radiation regarding sulfur containing amino acids [59]. 

**Figure 2:**
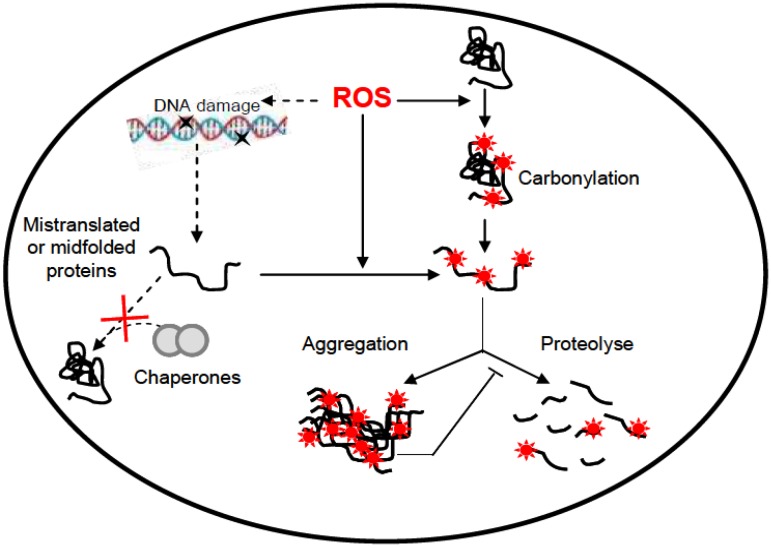
Formation of carbonylated proteins and their fate in the cell.

Carbonylation is one of the radiation-induced damage and is an irreversible oxidative process unlike methionine sulfoxide and cysteine disulfide bond formation [60]. Thus, a cell must rid itself of carbonylated proteins by degrading them. The Figure 2 illustrates the fate of damaged carbonylated proteins in the cell. Mistranslated proteins (due to DNA/RNA mutations) or otherwise mis-folded proteins (due to DNA/RNA mutations and/or chaperone deficiency) may become more susceptible to carbonylation (Figure 2) [61]. Proteomics demonstrated that this carbonylation is closely associated with the production of aberrant protein isoforms [62]. The rapid carbonylation of mistranslated or otherwise aberrant proteins points to an important physiological role of carbonylation in protein quality control. Since carbonylated proteins are more susceptible to proteolytic degradation than their non-oxidized counterparts [61,63,64,65], the rapid carbonylation of an erroneous protein may ensure that it is directed to the proteolyse process. Biochemical analysis revealed that carbonyl groups in the active center of a protein trigger its degradation [66,67]. Thus, carbonylation may act as a signal ensuring that damaged proteins enter the degradation pathway rather than the chaperone/repair pathway since carbonylation is an irreversible/unrepairable modification. However, highly carbonylated proteins can sometimes form high-molecular-weight aggregates that are proteolysis-resistant. Such aggregates appear to inhibit protease function (Figure 2) [62].

Carbonyl derivatives are mainly formed on the amino-acid side chains of proline, arginine, lysine, and threonine [68] and can also be formed by secondary reactions with reactive carbonyl compounds on carbohydrates (glycoxidation products), lipids, and advanced glycation/lipoxidation end products [68]. In bacteria, it was recently demonstrated that oxidative damage is the cause, rather than a consequence of radiation-induced cell death. This was demonstrated for both *Escherichia coli* and the radiation resistant bacterium, *Deinococcus radiodurans*, where ionizing radiation resistance was dependent on the level of protection against protein carbonylation [69]. In this way, sensitive bacteria would sustain lethal level of protein damage at radiation doses that elicit relatively little DNA damage, and that extreme resistance in bacteria would be dependent on protein protection [12]. It was reported in *E. coli* that the cells with low concentrations of carbonyl products remain reproductively competent, whereas cells with a high carbonyl load become genetically dead (unculturable) [70]. Diverse vital cellular functions like transcription, translation apparatus, transport systems, amino acids synthesis and degradation, transport systems, TCA cycle, glycolysis, chaperone functions and catalase were found to be targeted by UVA radiation in *E. coli* [71] and this was recently confirmed in *P. angustum* [72].

Indeed, protein targets affected by carbonylation in *P. angustum*, grown under UVB radiation, were alcohol dehydrogenase E, elongation factors (EF, Tu, NusA), chaperonins (GroEL, DnaK), ATPase, DNA gyrase, DNA directed RNA polymerases, and outer membrane protein (OmpL), the same than the ones found to be carbonylated in *E. coli* when cells were submitted to different stress conditions such as hydrogen peroxide, superoxide-generating compounds, and iron excess [73]. Proteins involved in metabolism, transcription, transport/folding and protein synthesis may therefore be the cellular functions that are most often affected by stress induced carbonylation, at least in certain bacteria. However, at present it is not clear what fraction of the vulnerable enzymes becomes modified during oxidative stress in different bacterial species, and whether such modifications typically interfere with protein function. This information is necessary in order to estimate the impact that this damage might have on cell viability.

## 7. Conclusion

Combined transcriptomic and proteomics studies suggested that the resistance to solar radiation result from a combination of different molecular mechanisms mainly due to an efficient DNA repair mechanism and an efficient protection against oxidative damage. However, the extreme resistance phenotype of *Deinococcus* species is still not fully understood. It should be noted that several hypothetical proteins must be functionally characterized to further explore the mechanisms of resistance of key bacterial species; members of this pool of proteins could have some remarkably interesting roles. The radioresistance of extreme radiotolerant bacteria is instructive in many aspects. Indeed, extremely efficient protection/repair mechanisms could be of special interest for broader applications such as cosmetic formulation (sunscreen, anti-aging), cancer and aging-related diseases, from which oxidative stress is tightly linked to, or for bioremediation of contaminated sites with radionucleides. Today, in a context of climate change with increasing levels of UVB radiation reaching the Earth’s surface, the knowledge of the impact of UV radiation on marine bacterial distribution, activity and gene regulation, is essential for understanding/predicting the possible alteration of biogeochemical cycling of elements in marine surface layers. 
